# Longitudinal assessment of the association between implementation strategy use and the uptake of hepatitis C treatment: Year 2

**DOI:** 10.1186/s13012-019-0881-7

**Published:** 2019-04-08

**Authors:** Shari S. Rogal, Vera Yakovchenko, Thomas J. Waltz, Byron J. Powell, Rachel Gonzalez, Angela Park, Maggie Chartier, David Ross, Timothy R. Morgan, JoAnn E. Kirchner, Enola K. Proctor, Matthew J. Chinman

**Affiliations:** 1 Center for Health Equity Research and Promotion, VA Pittsburgh Healthcare System, University Drive, Pittsburgh, PA USA; 2 Department of Surgery, University of Pittsburgh, Pittsburgh, PA USA; 3 Division of Gastroenterology, Hepatology, and Nutrition, University of Pittsburgh, Pittsburgh, PA USA; 4 Center for Healthcare Organization and Implementation Research, Edith Norse Rogers Memorial VA Hospital, Bedford, MA USA; 5 Department of Psychology, Eastern Michigan University, Ypsilanti, MI USA; 6 VA Center for Clinical Management Research, VA Ann Arbor Healthcare System, Ann Arbor, MI USA; 7 Department of Health Policy and Management, Gillings School of Global Public Health, University of North Carolina at Chapel Hill, Chapel Hill, NC USA; 8 Gastroenterology Section, VA Long Beach Healthcare System, Long Beach, CA USA; 9 Office of Strategic Integration | Veterans Engineering Resource Center, Washington, DC USA; 10 HIV, Hepatitis and Related Conditions Programs, Office of Specialty Care Services, Veterans Health Administration, Washington, DC USA; 11 Department of Veterans Affairs Medical Center, HSR&D and Behavioral Health Quality Enhancement Research Initiative (QUERI), Central Arkansas Veterans Healthcare System, Little Rock, AR USA; 12 Brown School, Washington University in St. Louis, St. Louis, MO USA; 13 RAND Corporation, Pittsburgh, PA USA

**Keywords:** Learning collaborative, Quality improvement, Cirrhosis, Advanced liver disease, Implementation science

## Abstract

**Background:**

To increase the uptake of evidence-based treatments for hepatitis C (HCV), the Department of Veterans Affairs (VA) established the Hepatitis Innovation Team (HIT) Collaborative. Teams of providers were tasked with choosing implementation strategies to improve HCV care. The aim of the current evaluation was to assess how site-level implementation strategies were associated with HCV treatment initiation and how the use of implementation strategies and their association with HCV treatment changed over time.

**Methods:**

A key HCV provider at each VA site (*N* = 130) was asked in two consecutive fiscal years (FYs) to complete an online survey examining the use of 73 implementation strategies organized into nine clusters as described by the Expert Recommendations for Implementing Change (ERIC) study. The number of Veterans initiating treatment for HCV, or “treatment starts,” at each site was captured using national data. Providers reported whether the use of each implementation strategy was due to the HIT Collaborative.

**Results:**

Of 130 sites, 80 (62%) responded in Year 1 (FY15) and 105 (81%) responded in Year 2 (FY16). Respondents endorsed a median of 27 (IQR19–38) strategies in Year 2. The strategies significantly more likely to be chosen in Year 2 included tailoring strategies to deliver HCV care, promoting adaptability, sharing knowledge between sites, and using mass media. The total number of treatment starts was significantly positively correlated with total number of strategies endorsed in both years. In Years 1 and 2, respectively, 28 and 26 strategies were significantly associated with treatment starts; 12 strategies overlapped both years, 16 were unique to Year 1, and 14 were unique to Year 2. Strategies significantly associated with treatment starts shifted between Years 1 and 2. Pre-implementation strategies in the “training/educating,” “interactive assistance,” and “building stakeholder interrelationships” clusters were more likely to be significantly associated with treatment starts in Year 1, while strategies in the “evaluative and iterative” and “adapting and tailoring” clusters were more likely to be associated with treatment starts in Year 2. Approximately half of all strategies were attributed to the HIT Collaborative.

**Conclusions:**

These results suggest that measuring implementation strategies over time is a useful way to catalog implementation of an evidence-based practice over time and across settings.

**Electronic supplementary material:**

The online version of this article (10.1186/s13012-019-0881-7) contains supplementary material, which is available to authorized users.

## Background

Hepatitis C virus (HCV) is a leading cause of liver cancer and liver failure in the USA [1]. In fiscal year 2015 (FY15), new, highly-efficacious treatments for HCV became widely available as the evidence-based practice for curing HCV [2]. Prior treatments included injected interferon, which was suboptimal because of side effects, contraindications, and poor efficacy despite year-long treatments. The newer medications included pill-only regimens with minimal side effects, short courses, and high cure rates. As the largest provider for HCV nationally, the Department of Veterans Affairs (VA) sought to spread this innovation rapidly across the country by developing the Hepatitis C Innovation Team (HIT) Collaborative. Funded by VA leadership as a 4-year, national initiative, the HIT Collaborative supported the development of regional teams of providers with the goal of promoting the uptake of evidence-based HCV care throughout the VA. The HIT Collaborative included the components of learning or quality improvement collaboratives [3], such as using in-person learning sessions, plan-do-study-act cycles, team calls, email/web-support, external support of active data collection, feedback and education by experts and Collaborative leadership, and outreach to local and national leadership.

Together, the availability of new HCV treatments and VA’s implementation efforts to increase their uptake resulted in a dramatic increase in treatment and cure of HCV in VA. While only 10% of Veterans with HCV infection had *ever* been cured of HCV as of the end of FY14, by the end of FY16, 43% or 84,192 Veterans were cured, representing a fourfold increase [4].

While this rapid, national implementation effort has been a tremendous success for VA, it has also provided the opportunity to study the use of implementation strategies and their association with a measurable clinical outcome over time and on a national scale. We previously reported on the associations between implementation strategies and HCV treatment starts at the site level and the extent to which strategies were related to HIT activities in the first year of the HIT Collaborative [5]. To frame our evaluation, we used expert-based definitions of implementation strategies, or methods to increase the uptake of evidence-based practices [6, 7], from the Expert Recommendations for Implementing Change (ERIC) project. ERIC defined 73 individual strategies [8] and then used a mixed-methods process called concept mapping [9] to develop conceptually distinct clusters of the strategies [10]. As the Collaborative continued, we had the opportunity to study how implementation strategy use changed over time within a nationwide healthcare system, particularly in the context of a learning collaborative [3].

This evaluation aimed to document (1) how reported implementation strategy use evolved over the first two years of the HIT Collaborative, (2) the changes in the associations between implementation strategies and clinical outcomes over time, and (3) the role of the HIT Collaborative in implementation strategy uptake.

## Methods

### Assessment of implementation strategies

Within VA, the HIT Collaborative was led by the National Hepatitis C Resource Center and the Office of Strategic Integration | Veterans Engineering Resource Center with the support of the National HIV, Hepatitis, and Related Conditions (HHRC) Program Office. These data were collected in service of the HIT Collaborative program evaluation, which was reviewed by the VA Pittsburgh Healthcare System IRB and deemed to be a quality improvement project and approved as such by HHRC. All participation in the evaluation was voluntary.

Using implementation strategies as defined by the ERIC project [8] and the clusters of strategies developed by Waltz et al. [10], we created a survey as previously described [5]. The survey asked whether each of the 73 strategies was used to improve HCV care at the site (yes/no) and, if so, whether the use of each strategy could be attributed to support provided by the HIT Collaborative (yes/no). We emailed providers a link to a web-based survey annually in FY15 (Year 1) and FY16 (Year 2).

### Recruitment

The HIT Collaborative provided the contact information for VA HCV providers and HIT Collaborative members (as listed on the self-provided team rosters) from the 130 VA medical “stations” as classified by Population Health Services of the VA [11]. The individuals who were emailed included providers with varying degrees of affiliation with the HIT Collaborative. Potential participants were emailed twice as a group and once individually by the HIT Collaborative Leadership team following a modified Dillman approach [12]. Additionally, the HIT Collaborative Leadership Team encouraged members to complete the assessment on regularly-scheduled calls.

At sites with more than one respondent, we retained a single response following a “key informant” technique, where a knowledgeable individual answers questions for a site [13]. In the first year, we determined that the responses would be preferentially retained from an HCV lead clinician. If this person did not respond, then we prioritized responses from the following providers (in descending order of priority): physician, pharmacist, nurse practitioner, physician assistant, other providers, and system redesign staff. In the second year, we prioritized retention from the repeat respondents. If there was not a response from the same person in the second year, then we followed the prioritization scheme as outlined above. Previous experience with the survey and discussions with HIT team members suggested that any of the individuals mentioned above would be knowledgeable enough to answer questions about HCV treatment and the use of implementation strategies.

### Data collection

In addition to collecting site-level implementation strategies in each year, respondents provided information regarding their participation in or affiliation with the HIT Collaborative (members vs. non-members), years in VA, and clinical specialty. Additionally, we classified sites using VA site complexity classifications [14]. These ratings range from levels 1a, 1b, 1c, 2, and 3, in descending order of complexity, and are based on site financial resources, number of patients served, acuity, and services provided. The primary clinical outcome of interest was the number of Veterans started on HCV treatment per year at each site, as defined by VA’s Population Health website [11].

### Analysis

We first described the provider and site characteristics in each year. For sites with more than one respondent in a given year, we calculated the interrater reliability. We then assessed the endorsement of strategies to determine which strategies were the most commonly used in Year 2 and the change in strategy use between years. We used chi-square tests to assess the statistical significance of the change in the proportion of participants using each strategy between years. The association between the total number of strategies and the total number of treatment starts was assessed using Pearson’s correlation and then linear regression, controlling for site complexity. Next, we assessed which individual strategies were significantly associated with the number of treatment starts using Spearman’s test of correlation. Using the map of strategy clusters from Waltz et al. [10], we arrayed the strategies significantly associated with treatment starts in Years 1 and 2 to show how they differed over time. Sensitivity analyses were conducted to assess whether the findings differed between repeat responders and first-time responders in Year 2 (at the site and individual respondent levels). We also assessed differences in responses by HIT membership status using chi-square tests.

For each implementation strategy, we asked participants whether they would attribute their use of the strategy at their site to the HIT Collaborative. We assessed these data by dividing the total number of sites attributing their use of a strategy to the HIT collaborative by the total number of sites endorsing that strategy. We then calculated the proportion of strategies endorsed in each cluster that was attributed to the HIT Collaborative.

## Results

### Respondent characteristics

In Year 1 (FY15) and Year 2 (FY16), 62% and 81% of 130 VA sites responded to the surveys, respectively. Of these sites, 69 (53%) responded in both years. The same individual responded in both years in 47 (36%) of these cases. In Year 2, 23 sites had duplicate responses, and the interrater reliability was 0.65. There were 11 sites that only responded in Year 1 and 34 sites that only responded in Year 2. The responding sites in Year 2 were responsible for 84% of all national HCV treatment starts in that year.

Table 1 shows the respondent characteristics in both years. While there was a trend towards more pharmacy providers and less primary care providers who responded in Year 2 vs. Year 1, this difference was not statistically significant (*p* = 0.14). Otherwise, the general demographic characteristics of the respondents were the same between years. There was a broad distribution of site complexity represented in both years. Notably, not all respondents were affiliated with members of the HIT Collaborative.

**Table 1:** Respondent characteristics

	Year 1 (FY15)		Year 2 (FY16)	
Characteristic	*N*	%	*N*	%
Number of sites (of 130 total)	80	62	105	81
HIT members	68	85	95	90
Years in VA				
< 3	13	16	23	22
4 to 9	25	31	31	30
10 to 19	25	31	38	36
> 20	17	21	13	12
Specialty				
Gastroenterology	33	41	42	40
Hepatology				
Infectious disease	17	21	21	20
Pharmacy	13	16	31	30
Primary care	8	10	6	6
Other (VERC, transplant)	9	11	5	5
Site complexity				
1a	27	33	34	32
1b	14	18	15	14
1c	12	15	16	15
2	14	18	19	18
3	12	15	21	20

The number of patients with HCV and the numbers and percentages treated in each year are illustrated in Table 2. Approximately 20% of patients in the participating sites were treated in Year 1.

**Table 2:** HCV treatment among VA site and responding sites

	Responding VA sites	
	Year 1 (*N* = 80)	Year 2 (*N* = 105)
Number of viremic veterans		
Total in all sites	103,991	112,935
Range	47 to 4243	38 to 3415
Median (n, IQR)	1149 (624, 1759)	935 (523, 1467)
HCV treatment starts		
Total (*n*)	20,503	31,821
Range (*n*)	3 to 1044	4 to 810
Median (*n*, IQR)	197 (124, 312)	264 (145, 416)
% Treated		
Total (treated/viremic)	20%	28%
Range (%)	6 to 47	7 to 60
Median (%)	18 (15, 24)	29 (24, 34)

### Association between the total number of strategies endorsed and treatment starts

The FY15 findings were previously published [5] and are presented here for comparison with the FY16 data. A mean of 25 ± 14 strategies were endorsed in Year 1 and 28 ± 14 strategies in Year 2. The total number of strategies endorsed was significantly correlated with the number of treatment starts in both years (Year 1 *r* = 0.43, *p* < 0.01; Year 2 *r* = 0.33, *p* < 0.01). The sites in the highest vs. lowest quartile of treatment starts endorsed significantly more strategies in both years (Year 1, 33 vs. 15 strategies; Year 2, 34 vs. 20, *p* < 0.01). The total number of strategies endorsed was significantly associated with total treatment starts when controlling for site complexity in both years. The adjusted *R*^2^ for these models was 0.30 in Year 1 and 0.29 in Year 2.

### Specific strategies endorsed in each year

The most commonly used strategies in both years were changing the record system, having the medications on the formulary, using data experts, data warehousing, tailoring strategies, promoting adaptability, engaging patients to be active participants in their care, and intervening with patients to promote uptake/adherence (Table 3). Overall strategy use was largely consistent between the two years; however, there were four strategies with statistically significant differential uptake. Those with increased uptake from FY15 to FY16 were tailoring strategies to deliver HCV care (+ 18%), promoting adaptability (+ 20%), sharing knowledge (+ 19%), and using mass media (+ 18%). None of the year-to-year decreases met the threshold for significance. At a more categorical level, the evaluative and iterative strategies had the least amount of change between the years, and the strategies in the clusters of engaging consumers and adapting and tailoring to the context had the most positive increases between the two years.

**Table 3:** Strategy endorsement in each year and change between years

#	Strategy and Cluster	Year 1 *N* = 80	Year 2 *N* = 105	Change
	Infrastructure			
1	• Change physical structure and equipment	53%	51%	− 2%
2	• Change the record systems	71%	57%	− 14%
3	• Change the location of clinical service sites	26%	37%	11%
4	• Develop a separate organization or group responsible for disseminating HCV care	23%	33%	10%
5	• Mandate changes to HCV care	55%	52%	− 3%
6	• Create or change credentialing and/or licensure standards	29%	30%	1%
7	• Participate in liability reform efforts that make clinicians more willing to deliver the clinical innovation	4%	11%	7%
8	• Change accreditation or membership requirements	4%	1%	− 3%
	Financial			
9	• Access new funding	30%	41%	11%
10	• Alter incentive/allowance structures	5%	10%	5%
11	• Provide financial disincentives for failure to implement or use the clinical innovations	0%	2%	2%
12	• Respond to proposals to deliver HCV care	44%	51%	7%
13	• Change billing	11%	14%	3%
14	• Place HCV medications on the formulary	70%	69%	− 1%
15	• Alter patient fees	0%	0%	0%
16	• Use capitated payments	0%	1%	1%
17	• Use other payment schemes	5%	2%	− 3%
	Support clinicians			
18	• Create new clinical teams	46%	50%	4%
19	• Facilitate the relay of clinical data to providers	56%	68%	12%
20	• Revise professional roles	50%	55%	5%
21	• Develop reminder systems for clinicians	34%	44%	10%
22	• Develop resource sharing agreements	26%	35%	9%
	Provide interactive assistance			
23	• Use outside assistance often called “facilitation”	8%	12%	4%
24	• Have someone from inside the clinic or center (often called “local technical assistance”) tasked with assisting the clinic	15%	25%	10%
25	• Provide clinical supervision	44%	48%	4%
26	• Use a centralized system to deliver facilitation	28%	28%	0%
	Adapt and tailor to context			
27	• Use data experts to manage HCV data	58%	70%	12%
28	• Use data warehousing techniques	85%	91%	6%
29	• Tailor strategies to deliver HCV care	63%	81%	18%*
30	• Promote adaptability	55%	75%	20%*
	Train and educate stakeholders			
31	• Conduct educational meetings	51%	64%	13%
32	• Have an expert in HCV care meet with providers to educate them	41%	53%	12%
33	• Provide ongoing HCV training	49%	60%	11%
34	• Facilitate the formation of groups of providers and fostered a collaborative learning environment	44%	43%	− 1%
35	• Developed formal educational materials	39%	35%	− 4%
36	• Distribute educational materials	55%	55%	0%
37	• Provide ongoing consultation with one or more HCV treatment experts	58%	71%	13%
38	• Train designated clinicians to train others	20%	26%	6%
39	• Vary the information delivery methods to cater to different learning styles when presenting new information	36%	36%	0%
40	• Give providers opportunities to shadow other experts in HCV	33%	22%	− 11%
41	• Use educational institutions to train clinicians	11%	15%	4%
	Develop stakeholder interrelationships			
42	• Build a local coalition/team to address challenges	53%	53%	0%
43	• Conduct local consensus discussions	48%	54%	6%
44	• Obtain formal written commitments from key partners that state what they will do to implement HCV care	4%	4%	0%
45	• Recruit, designate, and/or train leaders	26%	23%	− 3%
46	• Inform local opinion leaders about advances in HCV care	49%	46%	− 3%
47	• Share the knowledge gained from quality improvement efforts with other sites outside your medical center	38%	57%	19%*
48	• Identify and prepare champions	50%	52%	2%
49	• Organize support teams of clinicians who are caring for patients with HCV and given them time to share the lessons learned and support one another’s learning	26%	32%	6%
50	• Use advisory boards and interdisciplinary workgroups to provide input into HCV policies and elicit recommendations	26%	22%	− 4%
51	• Seek the guidance of experts in implementation	44%	50%	6%
52	• Build on existing high-quality working relationships and networks to promote information sharing and problem solving related to implementing HCV care	61%	71%	10%
53	• Use modeling or simulated change	13%	15%	2%
54	• Partner with a university to share ideas	14%	11%	− 3%
55	• Make efforts to identify early adopters to learn from their experiences	16%	24%	8%
56	• Visit other sites outside your medical center to try to learn from their experiences	15%	20%	5%
57	• Develop an implementation glossary	3%	6%	3%
58	• Involve executive boards	23%	33%	10%
	Use evaluative and iterative strategies			
59	• Assess for readiness and identify barriers and facilitators to change	26%	30%	4%
60	• Conduct a local needs assessment	45%	43%	− 2%
61	• Develop a formal implementation blueprint	34%	36%	2%
62	• Start with small pilot studies and then scale them up	23%	25%	2%
63	• Collect and summarize clinical performance data and give it to clinicians and administrators to implement changes in a cyclical fashion using small tests of change before making system-wide changes	21%	26%	5%
64	• Conduct small tests of change, measured outcomes, and then refined these tests	19%	21%	2%
65	• Develop and use tools for quality monitoring	41%	32%	− 9%
66	• Develop and organize systems that monitor clinical processes and/or outcomes for the purpose of quality assurance and improvement	30%	28%	− 2%
67	• Intentionally examine the efforts to promote HCV care	61%	69%	8%
68	• Develop strategies to obtain and use patient and family feedback	20%	20%	0%
	Engage consumers			
69	• Involve patients/consumers and family members	50%	61%	11%
70	• Engage in efforts to prepare patients to be active participants in HCV care	63%	57%	− 6%
71	• Intervene with patients/consumers to promote uptake and adherence to HCV treatment	71%	79%	8%
72	• Use mass media to reach large numbers of people	18%	36%	18%*
73	• Promote demand for HCV care among patients through any other means	40%	52%	12%

The bold and * represent statistically significant changes between years

Table 4 shows that the strategies significantly associated with HCV treatment changed across the two years and that the difference-making strategies varied by year. In Years 1 and 2, respectively, 28 and 26 strategies were significantly associated with treatment starts, 12 strategies overlapped both years, 16 were unique to Year 1, and 14 were unique to Year 2.

**Table 4:** Strategies significantly associated with treatment in both years vs. only Year 1 or Year 2

Both years	Year 1 only	Year 2 only
Change infrastructure		
• Change physical structure/equipment • Change the location of clinical service sites	• Change accreditation or membership requirements • Liability reform	• Change the record systems
Financial strategies		
		• Alter incentive/allowance structures
Support clinicians		
• Create new clinical teams • Revise professional roles	• Develop resource sharing agreements	• Facilitate the relay of clinical data to providers
Provide interactive assistance		
• Provide clinical supervision	• Local technical assistance • Use a centralized system to deliver facilitation	
Adapt and tailor to the context		
		• Use data experts to manage HCV data
Train/educate providers		
• Facilitate the formation of groups of providers and foster a collaborative learning environment	• Conduct educational meetings • Have an expert in HCV care meet with providers to educate them • Provide ongoing HCV training • Vary information delivery methods	• Use educational institutions to train clinicians • Distribute educational materials
Develop stakeholder interrelationships		
• Build a local coalition/team to address challenges • Conduct local consensus discussions • Recruit, designate, and/or train leaders • Use modeling or simulated change • Make efforts to identify early adopters to learn from their experiences	• Partner with a university • Visit other sites outside your medical center to try to learn from their experiences • Identify and prepare champions • Inform local opinion leaders • Share the knowledge gained from quality improvement efforts with other sites • Build on existing high-quality working relationships and networks to promote information sharing and problem solving	• Organize support teams of clinicians who are caring for patients with HCV and given them time to share the lessons learned and support one another’s learning • Involve executive boards
Use evaluative and iterative strategies		
• Collect and summarize clinical performance data and give it to clinicians and administrators to implement changes in a cyclical fashion using small tests of change before making system-wide changes		• Assess for readiness and identify barriers and facilitators to change • Develop a formal implementation blueprint • Develop and organize systems that monitor clinical processes and/or outcomes for the purpose of quality assurance and improvement • Intentionally examine the efforts to promote HCV care • Conduct small tests of change, measured outcomes, and then refined these tests • Develop strategies to obtain and use patient and family feedback
Engage consumers		
	• Engage in efforts to prepare patients to be active participants in HCV care	

**Fig. 1:**
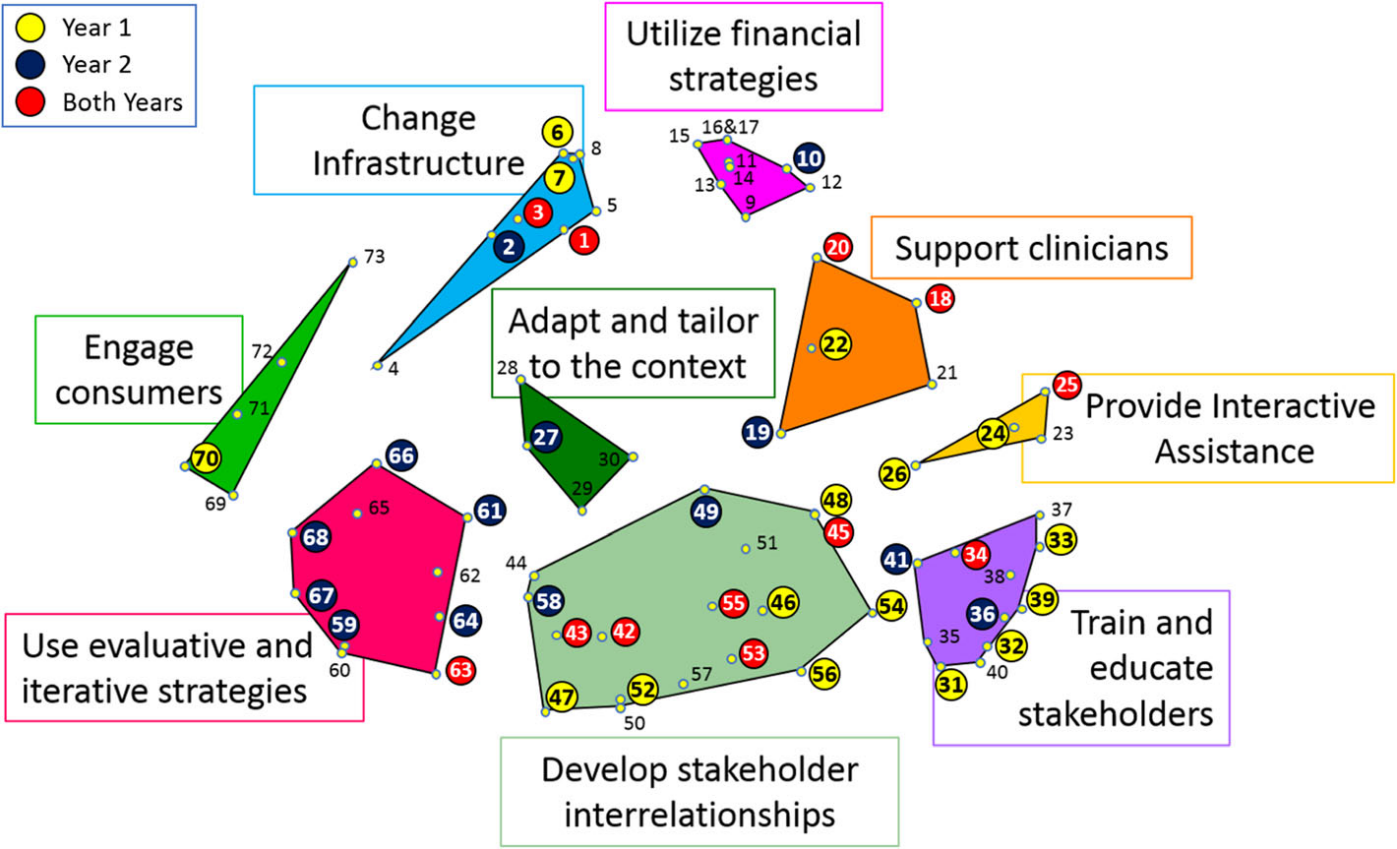
Strategies associated with treatment starts in Year 1 vs. Year 2 mapped onto strategy clusters

Figure 1 illustrates the changes in the strategies significantly associated with treatment starts unique to each year overlaid on the ERIC cluster map, with numbering corresponding to Table 2. The map shows that the significant strategies shifted locations between the years. In Year 1 of availability of the new clinical innovation, the uptake of treatment was significantly associated with strategies in the "provide interactive assistance", "train and educate stakeholders", and "develop stakeholder interrelationships" clusters. In Year 2, the significant strategies were in the "use evaluative and iterative strategies" and adapt and tailor to the context" clusters.

We assessed differences in strategy endorsement between repeat responders and new responders in Year 2. The sites that were newly responding in Year 2 had strategy endorsement patterns more similar to repeat responders’ responses in Year 2 than in Year 1. One exception is that in Year 2, newly responding sites were significantly more likely to endorse “change the record” system than repeat sites (72% vs. 49%, *p* = 0.01). Otherwise, strategy endorsement appeared very similar to that of the Year 2 results for sites that had also responded in Year 1.

Respondents who were participating in the HIT Collaborative were significantly more likely to endorse specific strategies (Table 5). The strategies associated with increased treatment starts are highlighted in bold. Eight of the 10 strategies that were more likely to be endorsed by HIT members in Year 1 were also significantly positively associated with treatment starts in that year. The two strategies that were more likely to be endorsed by HIT members in Year 2 were significantly associated with treatment starts in Year 1 but not Year 2.

**Table 5:** Strategies significantly associated with HIT membership*

Strategy	Non-HIT member endorsement (%)	HIT member endorsement (%)
**Year 1**	***N*** **= 12**	***N*** **= 68**
**• Conduct educational meetings**	**17%**	**57%**
**• Provide ongoing HCV training**	**17%**	**54%**
**• Conduct local consensus discussions**	**17%**	**53%**
**• Use a centralized system to deliver facilitation**	**0%**	**32%**
**• Share the knowledge gained from quality improvement efforts with other sites outside your medical center**	**8%**	**43%**
• Tailor strategies to deliver HCV care	33%	68%
**• Develop resource sharing agreements**	**0%**	**31%**
**• Build a local coalition/team to address challenges**	**25%**	**57%**
• Respond to proposals to deliver HCV care	17%	49%
**• Provide clinical supervision**	**17%**	**49%**
**Year 2**	***N*** **= 10**	***N*** **= 95**
• Inform local opinion leaders about advances in HCV care	82%	100%
• Identify and prepare champions	84%	96%

*Only strategies that were significantly associated with HIT membership are shown in this table; bolded strategies are those associated with treatment starts in that year

### Attribution to the HIT Collaborative

**Fig. 2:**
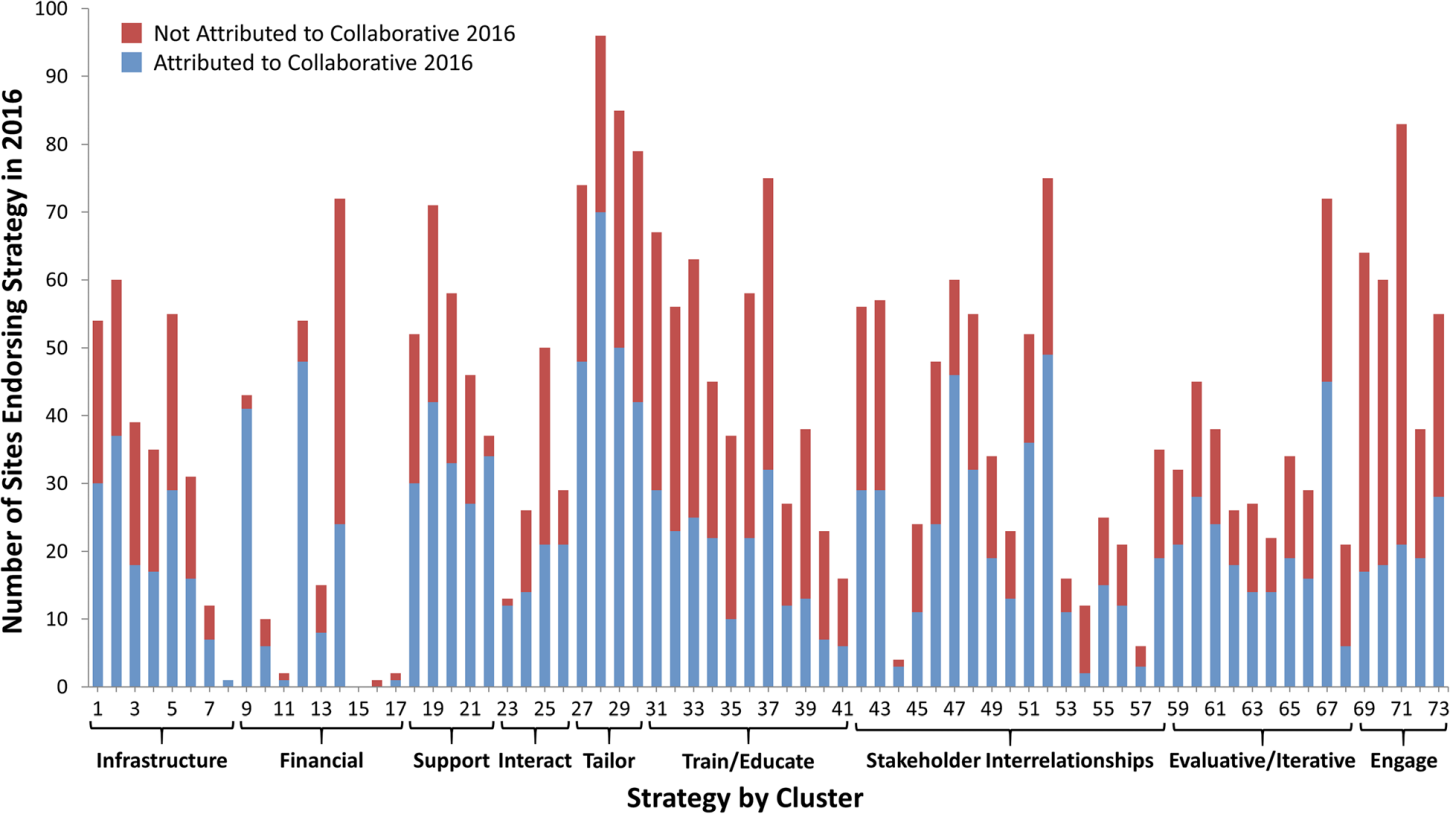
Strategy use and attribution to the HIT Collaborative in Year 2

Respondents self-reported whether each strategy they endorsed was used as a result of the HIT Collaborative (or would not have been used if it were not for the HIT Collaborative), and we assessed this in each year and how this changed over time (raw data presented in Additional file 1). Figure 2 shows the total number of sites endorsing each strategy (height of vertical bars) and the proportion that was attributed to the HIT Collaborative (blue). In Year 2, 54% of all strategy use was attributed to the HIT Collaborative, compared to 41% in Year 1. The ranges of strategy use and attribution were wide. Since the results were similar in both years, Year 2 (FY16) is presented below.

Table 6 shows the change between years of strategies attributed to the HIT Collaborative. The cluster least likely to be attributed to the Collaborative was “engaging consumers.” “Training and educating stakeholders” was also unlikely to be attributed to the HIT Collaborative in Year 1 (27%), but the percent attribution increased to 40% in Year 2. There was a 21% increase in the strategies attributable to the HIT in the "evaluative and iterative" cluster between the two years. HIT members were more likely than non-HIT members to attribute seeking the guidance of experts in implementation (29% vs. 9%, *p* = 0.01) and identifying and preparing champions (36% vs. 16%, *p* = 0.03) to the Collaborative.

**Table 6:** Percentage of strategies attributed to the HIT Collaborative by cluster in each year

Cluster	Percent of strategies attributed to HIT Collaborative		
	Year 1	Year 2	Change
Change infrastructure	48	54	6
Financial strategies	56	65	9
Support clinicians	57	63	6
Provide interactive assistance	40	58	18
Adapt and tailor to the context	58	63	5
Train and educate stakeholders	27	40	13
Develop stakeholder relationships	41	59	18
Use evaluative and iterative strategies	38	59	21
Engage consumer	20	34	14

## Discussion

We previously examined self-reported use of implementation strategies in a national sample and found that the total number of strategies used by a site was associated with the clinical outcome of HCV treatment starts [5]. In this study, we further investigated the use of strategies over time and the associations between site-level implementation strategy use and treatment starts over time. While many of the strategies did not change in use from Year 1 to Year 2, there was a significant increase in the following specific strategies: tailoring strategies, promoting adaptability, sharing knowledge, and using mass media. Moreover, while the total number of strategies used was associated with increased HCV treatment in each year, the specific strategies associated with treatment starts varied over time.

The EPIS Implementation Framework posits that implementation happens along four phases: Exploration, Preparation, Implementation, and Sustainment. The implementation strategies that are appropriate may vary by the stage of implementation [15]. These data support that the implementation strategies associated with successful implementation of a clinical innovation change over time. When the oral HCV medications/clinical innovation first became available, successful sites focused on preparation or “pre-implementation.” The associated implementation strategies included training and education, as well as developing stakeholder interrelationships and seeking interactive assistance. After sites had established the necessary education and relationships, the most successful sites then focused on iterating these and adapting to the context in Year 2. In other words, the strategies associated with treatment shifted across the ERIC group’s concept map between years. The geography of this concept map was developed by implementation experts considering the global similarities of the strategies. The present data suggest that clusters and strategies within clusters may be differentially relevant based on the phase(s) of implementation. For example, strategies from the “train and education stakeholders” and “develop stakeholder interrelationships” clusters were important in the first year while the strategies within the “use evaluative and iterative strategies” cluster were more closely associated with treatment starts in the second year. A more detailed accounting of the specific phases of implementation that were present over the reporting period could further clarify the relationships between phases of implementation and the strategies used. Our finding that the strategies associated with HCV treatment changed from Year 1 to Year 2 supports the notion that successful sites used evolving strategies as the clinical innovation became more available and as the learning collaborative evolved.

These results must be interpreted in the context of the national HIT Collaborative. The timing of the national efforts to improve HCV care also corresponded to a major shift in the treatment of HCV from difficult-to-use, interferon-based treatments to simple, highly efficacious, curative, pill-based treatments. We aimed to assess how the Collaborative influenced the choice of activities to promote HCV care and how much of the strategy uptake related to the HIT Collaborative itself versus independent, local activities in response to the availability of these newer HCV medications. We asked providers to comment on whether strategies would have been done without the Collaborative and assessed how members of the Collaborative employed strategies differently than non-members. We found that approximately half of all implementation efforts nationally were attributed to the HIT Collaborative, meaning that providers felt that half of the activities would not have been done without the HIT Collaborative. Moreover, the activities that members of the Collaborative were more likely to engage in were those that are considered to be core elements of learning/quality improvement collaboratives in the literature [3]. For example, education/training, team-building, and communication with leadership are all essential elements of learning collaboratives and were endorsed more frequently by HIT members than non-members. Thus, these analyses are useful in assessing the role of the learning collaborative in the uptake of the clinical innovation via implementation strategy uptake.

While learning collaboratives are increasingly popular, their effectiveness is relatively untested [3]. These data provide preliminary support for the effectiveness of learning collaboratives. For example, strategies that were difference-making in this sample were often those strategies considered to be core components of learning collaboratives including using data relay, training and education, creating new teams, facilitation/technical assistance, and stakeholder interrelationship strategies. Moreover, the ERIC strategy “facilitate the formation of groups of providers and foster a collaborative learning environment” which specifically refers to learning collaboratives was significantly associated with treatment in both years, suggesting that the learning collaborative itself was associated with increased treatment. The HIT Collaborative specifically focused on building stakeholder interrelationships and using principles of system redesign including rapid cyclic tests of change. Sites frequently endorsed these types of strategies and attributed their uptake to the Collaborative. Additionally, the HIT Collaborative attribution in the "evaluative and iterative" cluster, which was critical to the success in Year 2, increased substantially from Year 1 to 2, indicating that the Collaborative was instrumental to site-level success. Most of the strategies endorsed more often by HIT members vs. non-members were significantly associated with treatment starts. These data thus provide some preliminary support for learning collaboratives as an effective means of increasing the uptake of a clinical innovation or evidence-based practice.

Implementation strategies have historically been difficult to measure. Generally, tracking strategies has been completed by observation and not by self-reporting on a comprehensive list of strategies [16, 17]. While we previously reported on developing a survey using the definitions of implementation strategies from the ERIC study, it remained unclear whether these strategies would be understandably and reliably interpreted by non-implementation scientists. In both years of data collection, we found an association between clinical outcomes with specific implementation strategies. The second year of data collection further demonstrates that providers could interpret and answer questions about implementation strategies. First, there was adequate interrater reliability within sites in both years. Second, there was consistency across the years in that several of the strategies were associated with treatment starts. Third, the strategies that were individually associated with treatment starts were in some cases those strategies supported by implementation literature. For example, facilitation is a well-studied strategy and was associated with higher treatment rates in Year 1 [18–22]. Fourth, we found that the providers were able to generally distinguish between similar strategies. The strategy clusters were designed to group similar strategies, and we did not find strong correlations between endorsement of specific strategies within a cluster. In fact, there was significant variation in endorsement of the strategies within clusters (where the most similar strategies are housed). These findings indicate that such surveys can be used to track implementation strategies across a wide range of provider types, education, and geographic locations.

This study has several notable limitations. First, we relied on year-end self-report of implementation and included only one response per site. We found that the results had face-validity, as outlined above, and there was adequate interrater reliability when we assessed the reports of sites with more than one respondent. However, future studies would benefit from directly observing site-level implementation or documenting the application of implementation strategies over the course of the reporting time period. Second, it is unclear if the self-reported attribution data related to increased awareness of the HIT Collaborative or social desirability, given that more of the respondents in Year 2 were HIT members. Theoretically, any strategy could have been “correctly” attributed to the Collaborative, since the Collaborative leadership team encouraged and supported any strategy that the sites felt would be effective. A third limitation is that we included limited contextual factors in our associations between implementation strategy use and clinical outcomes. However, participant characteristics were not significantly associated with strategy endorsement. Given the uniformity of structure within VA, this may be less important, but in applying these lessons to non-VHA sites, more contextual information may have to be collected. We were also unable to assess the timing, sequencing, or intensity of implementation strategies within each year. It may be that specific strategies need to be sequenced in a specific order within the year. While the simple listing of strategies allowed us to quickly collect data from across the country, these data do not detail how the strategies were operationalized by sites. Often the application of implementation strategies may vary broadly and lead to difficulties assessing which elements of a strategy are critical or difference-making [23]. We successfully tracked strategies across the years, which is a strength of these analyses. A final limitation was our limited choice of outcome measures. We considered focusing on the proportion of patients treated as our primary outcome but were concerned that sites with fewer patients would have an artificial advantage, since sites treating half of the patients would look the same, whether they had 20 or 2000 patients to treat. Our findings should be validated in other clinical contexts with other medical problems. Future work in this area could aim to address whether specific combinations of strategies are important or how to use these data to address low-performing sites. Future work could also assess the associations between the sites’ stage of implementation [24] and strategy utilization.

## Conclusions

These findings collectively indicate that the strategies associated with the uptake of a clinical innovation change over time from “pre-implementation” strategies including training and education, interactive assistance, and developing stakeholder interrelationships to strategies that are evaluative and iterative and adapt to the context, which indicate a more mature phase of implementation. This research advances the field by providing support for the implementation strategies and clusters developed by the ERIC group. This project demonstrates the utility of deploying surveys of implementation strategies sequentially across the life of a national clinical program, which could provide guidance to other national initiatives.

## Additional file

Additional file 1: Participant attribution of strategies to the HIT Collaborative in each year. (DOCX 18 kb)

## Abbreviations

ERIC: Expert Recommendations for Implementing Change
FY: Fiscal year
HCV: Hepatitis C virus
HIT: Hepatitis C Innovation Team
IQR: Interquartile range
VA: Department of Veterans Affairs
