# Supplementary material for: Longitudinal assessment of the association between implementation strategy use and the uptake of hepatitis C treatment: Year 2
Source: Implement Sci. 2019 Apr 8;14:36. doi: 10.1186/s13012-019-0881-7 (PMC6454775; doi:10.1186/s13012-019-0881-7)
Supplement: Supplementary file 1 — Participant attribution of strategies to the HIT Collaborative in each year. (DOCX 18 kb) [file 13012_2019_881_MOESM1_ESM.docx]

**Additional file 1: Participant Attribution of Strategies to the HIT Collaborative in Each Year**

| Strategy | Year 1 Attribution  # (%) | Year 2 Attribution  # (%) | Change |
| --- | --- | --- | --- |
| • Change physical structure/equipment | 19 (45) | 30 (56) | 11% |
| • Change record systems | 31 (54) | 37 (62) | 8% |
| • Change location of clinical service sites | 11 (52) | 18 (46) | -6% |
| • Develop a separate group responsible for disseminating HCV care | 8 (44) | 17 (49) | 5% |
| • Mandate changes to HCV care | 23 (52) | 29 (53) | 1% |
| • Create or change credentialing and/or licensure standards | 6 (26) | 16 (52) | 26% |
| • Participate in liability reform efforts | 2 (67) | 7 (58) | -9% |
| • Change accreditation/membership requirements | 2 (67) | 1 (100) | 33% |
| • Access new funding | 24 (100) | 41 (95) | -5% |
| • Alter incentive/allowance structures | 2 (50) | 6 (60) | 10% |
| • Provide financial disincentives | 0 (0) | 1 (50) | 50% |
| • Respond to proposals to deliver HCV care | 29 (83) | 48 (89) | 6% |
| • Change billing | 3 (33) | 8 (53) | 20% |
| • Place HCV medications on the formulary | 15 (27) | 24 (33) | 6% |
| • Alter patient fees | 0 (0) | 0 (0) | 0% |
| • Use capitated payments | 0 (0) | 0 (0) | 0% |
| • Use other payment schemes | 1 (25) | 1 (50) | 25% |
| • Create new clinical teams | 17 (46) | 30 (58) | 12% |
| • Facilitate the relay of clinical data to providers | 28 (62) | 42 (59) | -3% |
| • Revise professional roles | 18 (45) | 33 (57) | 12% |
| • Develop reminder systems for clinicians | 16 (59) | 27 (59) | 0% |
| • Develop resource sharing agreements | 18 (86) | 34 (92) | 6% |
| • Use outside assistance often called “facilitation” | 4 (67) | 12 (92) | 25% |
| • Local technical assistance | 3 (25) | 14 (54) | 29% |
| • Provide clinical supervision | 8 (23) | 21 (42) | 19% |
| • Use a centralized system (i.e., from the VISN) to deliver facilitation | 15 (68) | 21 (72) | 4% |
| • Use data experts to manage HCV data | 29 (63) | 48 (65) | 2% |
| • Use data warehousing techniques | 48 (71) | 70 (73) | 2% |
| • Tailor strategies to deliver HCV care | 24 (48) | 50 (59) | 11% |
| • Promote adaptability | 20 (45) | 42 (53) | 8% |
| • Conduct educational meetings | 15 (37) | 29 (43) | 6% |
| • Have an expert in HCV care meet with providers to educate them | 10 (30) | 23 (41) | 11% |
| • Provide ongoing HCV training | 11 (28) | 25 (40) | 12% |
| • Facilitate the formation of groups of providers and fostered a collaborative learning environment | 9 (26) | 22 (49) | 23% |
| • Developed formal educational materials | 7 (23) | 10 (27) | 4% |
| • Distribute educational materials | 12 (27) | 22 (38) | 11% |
| • Provide ongoing consultation with one or more HCV treatment experts | 14 (30) | 32 (43) | 13% |
| • Train designated clinicians to train others | 5 (31) | 12 (44) | 13% |
| • Vary the information delivery methods to cater to different learning styles when presenting new information | 4 (14) | 13 (34) | 20% |
| • Give providers opportunities to shadow other experts in HCV | 6 (23) | 7 (30) | 7% |
| • Use educational institutions to train clinicians | 2 (22) | 6 (38) | 16% |
| • Build a local coalition/team to address challenges | 16 (38) | 29 (52) | 14% |
| • Conduct local consensus discussions | 10 (26) | 29 (51) | 25% |
| • Obtain formal written commitments from key partners that state what they will do to implement HCV care | 1 (33) | 3 (75) | 42% |
| • Recruit, designate, and/or train leaders | 11 (52) | 11 (46) | -6% |
| • Inform local opinion leaders about advances in HCV care | 13 (33) | 24 (50) | 17% |
| • Share the knowledge gained from quality improvement efforts with other sites outside your medical center | 18 (60) | 46 (77) | 17% |
| • Identify and prepare champions | 16 (40) | 32 (58) | 18% |
| • Organize support teams of clinicians who are caring for patients with HCV and given them time to share the lessons learned and support one another’s learning | 5 (24) | 19 (56) | 32% |
| • Use advisory boards and interdisciplinary workgroups to provide input into HCV policies and elicit recommendations | 9 (43) | 13 (57) | 14% |
| • Seek the guidance of experts in implementation | 18 (51) | 36 (69) | 18% |
| • Build on existing high-quality working relationships and networks to promote information sharing and problem solving related to implementing HCV care | 26 (53) | 49 (65) | 12% |
| • Use modeling or simulated change | 5 (50) | 11 (69) | 19% |
| • Partner with a university to share ideas | 1 (9) | 2 (17) | 8% |
| • Make efforts to identify early adopters to learn from their experiences | 7 (54) | 15 (60) | 6% |
| • Visit other sites outside your medical center to try to learn from their experiences | 6 (50) | 12 (57) | 7% |
| • Develop an implementation glossary | 0 (0) | 3 (50) | 50% |
| • Involve executive boards | 4 (22) | 19 (54) | 32% |
| • Assess for readiness and identify barriers and facilitators to change | 6 (29) | 21 (66) | 37% |
| • Conduct a local needs assessment | 15 (42) | 28 (62) | 20% |
| • Develop a formal implementation blueprint | 15 (56) | 24 (63) | 7% |
| • Start with small pilot studies and then scale them up | 6 (33) | 18 (69) | 36% |
| • Collect and summarize clinical performance data and give it to clinicians and administrators to implement changes in a cyclical fashion using small tests of change before making system-wide changes | 6 (35) | 14 (52) | 17% |
| • Conduct small tests of change, measured outcomes, and then refined these tests | 7 (47) | 14 (64) | 17% |
| • Develop and use tools for quality monitoring (this includes standards, protocols and measures to monitor quality) | 14 (42) | 19 (56) | 14% |
| • Develop and organize systems that monitor clinical processes and/or outcomes for the purpose of quality assurance and improvement | 10 (42) | 16 (55) | 13% |
| • Intentionally examine the efforts to promote HCV care | 16 (33) | 45 (63) | 30% |
| • Develop strategies to obtain and use patient and family feedback | 3 (19) | 6 (29) | 10% |
| • Involve patients/consumers and family members | 5 (13) | 17 (27) | 14% |
| • Engage in efforts to prepare patients to be active participants in HCV care | 10 (20) | 18 (30) | 10% |
| • Intervene with patients/consumers to promote uptake and adherence to HCV treatment | 11 (19) | 21 (25) | 6% |
| • Use mass media | 3 (21) | 19 (50) | 29% |
| • Promote demand for HCV care among patients through any other means | 9 (28) | 28 (51) | 23% |
